# The Relation of Phase‐Transition Effects and Thermal Stability of Planar Perovskite Solar Cells

**DOI:** 10.1002/advs.201801079

**Published:** 2018-10-25

**Authors:** Chuanjiang Qin, Toshinori Matsushima, Dino Klotz, Takashi Fujihara, Chihaya Adachi

**Affiliations:** ^1^ Center for Organic Photonics and Electronics Research (OPERA) Kyushu University 744 Motooka Nishi Fukuoka 819‐0395 Japan; ^2^ Japan Science and Technology Agency (JST) ERATO Adachi Molecular Exciton Engineering Project 744 Motooka Nishi Fukuoka 819‐0395 Japan; ^3^ International Institute for Carbon Neutral Energy Research (WPI‐I2CNER) Kyushu University 744 Motooka Nishi Fukuoka 819‐0395 Japan; ^4^ Innovative Organic Device Laboratory Institute of Systems Information Technologies and Nanotechnologies (ISIT) 744 Motooka Nishi Fukuoka 819‐0395 Japan

**Keywords:** impedance, perovskite alloys, perovskite solar cells, phase transition, stability

## Abstract

A power conversion efficiency of over 20% has been achieved in CH_3_NH_3_PbI_3_‐based perovskite solar cells (PSC), however, low thermal stability associated with the presence of a phase transition between tetragonal and cubic structures near room temperature is a major issue that must be overcome for future practical applications. Here, the influence of the phase transition on the thermal stability of PSCs is investigated in detail by comparing four kinds of perovskite films with different compositions of halogen atoms and organic components. Thermally stimulated current measurements reveal that a large number of carrier traps are generated in solar cells with the perovskite CH_3_NH_3_PbI_3_ as a light absorber after operation at 85 °C, which is higher than the phase‐transition temperature. Electrochemical impedance spectroscopy measurements further exclude effects of a possible morphology change on the formation of carrier traps. These carrier traps are detrimental to the thermal stability. The thermogravimetric analysis does not show a decomposition for any of the materials in the temperature range relevant for operation. The perovskite alloys do not have this phase transition, resulting in effectively suppressed formation of carrier traps. PSCs with improved thermal stability under the standard thermal cycling test are demonstrated.

Organic–inorganic halide perovskite solar cells (PSCs) are promising for next‐generation clean energy because they can achieve high power conversion efficiencies (η) and be fabricated with simple and low‐cost methods.^1^, ^2^ Although a certified η of 22.1% has been achieved through state‐of‐the‐art device engineering, the device stability is still not sufficient for widespread commercialization.^3^, ^4^, ^5^ Extrinsic factors such as moisture, oxygen, UV light, and temperature are known to limit the stability of perovskite materials and PSCs made from them,^5^, ^6^, ^7^, ^8^, ^9^ but intrinsic degradation mechanisms must still be further clarified to find new solutions for fabricating PSCs with excellent long‐term stability.

Among the known intrinsic factors, carrier traps and defects that degrade the device performance and lifetime have already been observed in PSCs with methylammonium lead triiodide (CH_3_NH_3_PbI_3_) perovskite absorbers.^10^, ^11^, ^12^ Several possible origins of defects have been proposed based on different experimental techniques and theoretical simulations.^13^, ^14^, ^15^ Stevart et al. reported that the chemical equilibria among iodoplumbate species may play a role in the creation of charge recombination centers.^14^ By decreasing the concentration of the lead ion species used during perovskite film formation, the concentration of defect precursor could be reduced in solutions. In our work, we found that hole traps are easily generated after exposure of CH_3_NH_3_PbI_3_ perovskite films to the moisture in air, which accelerates the degradation of PSCs under continuous light irradiation.^12^ Through systematic experimental studies, we showed that Frenkel defects are detrimental to the stability of PSCs and that metallic lead related vacancies are a possible intrinsic origin of carrier traps.^15^ By virtue of the weak reduction properties of a benzoquinone additive, we were able to suppress the formation of metallic lead and effectively extend the lifetime of the PSCs.

Another important consideration is that phase transitions exist in most perovskite materials. For example, the widely used CH_3_NH_3_PbI_3_ has two phase transitions: one is the phase transition between the octahedral structure and the tetragonal structure at around 161 K and the other is the phase transition between the tetragonal structure and the cubic structure at around 328 K.^16^ Based on the analysis of thermally stimulated current (TSC), the phase transition at around 161 K has been shown to induce the formation of carrier traps.^11^, ^12^, ^15^ Since this phase‐transition temperature is much lower than the operating temperature of PSCs in naturally occurring terrestrial environments, the influence of the low‐temperature phase transition on device performance should be negligible. However, the high‐temperature phase transition is at a temperature just slightly higher than room temperature.^17^ Therefore, how the high‐temperature phase transition affects the device performance and stability must be understood to develop PSCs that can pass strict lifetime tests under high temperatures up to 85 °C for future practical applications.

Here, we first correlate the phase transition with device properties such as conversion efficiency, degradation behavior, and lifetime at 85 °C for four kinds of lead‐based perovskite materials with different combinations of halogens (bromine (Br) and iodine (I)) and organic components (methylammonium (MA) and formamidinium (FA)). The chemical formulas of the perovskites used in this study are MA_0.6_FA_0.4_PbI_3_, MAPbI_2.6_Br_0.4_, MA_0.6_FA_0.4_PbI_2.8_Br_0.2_, and MAPbI_3_. Among them, the perovskite alloys (MA_0.6_FA_0.4_PbI_3_, MAPbI_2.6_Br_0.4_, and MA_0.6_FA_0.4_PbI_2.8_Br_0.2_) do not exhibit a phase transition in the temperature range from room temperature to 200 °C while the pure perovskite MAPbI_3_ has a phase transition at 54.6 and 56.2 °C for the exothermic and endothermic processes, respectively, as measured by differential scanning calorimetry (DSC). Furthermore, PSCs utilizing the pure perovskite and perovskite alloys as the light absorber demonstrated different degradation behavior under continuous light irradiation at 85 °C.^18^ Among the PSCs tested in this study, the MA_0.6_FA_0.4_PbI_2.8_Br_0.2_‐based PSCs achieved the best thermal stability due to reduced carrier trap formation as confirmed by TSC and impedance analysis.

Since perovskite alloys have mainly been used in mesoporous solar cells with the perovskite alloy infiltrating a semiconductor scaffold,^19^, ^20^, ^21^ we first screened several types of perovskite materials, MA_0.6_FA_0.4_PbI_3_, MAPbI_2.6_Br_0.4_, MA_0.6_FA_0.4_PbI_2.8_Br_0.2_, and MAPbI_3_, to find the optimal components for high‐performance planar devices. The spin‐coated perovskite‐alloy films fabricated here have absorption characteristics similar to what has been previously reported:^19^, ^20^ introduction of FA or Br into MAPbI_3_ to yield MA_0.6_FA_0.4_PbI_3_ and MAPbI_2.6_Br_0.4_ causes the absorption onset to shift to the red or blue, respectively, as shown in **Figure** **1**a. Because we optimized spin‐coating conditions, such as the molar ratios of each component in the precursor solutions, toluene‐dropping timing, and thermal annealing temperature and duration, for each film, all of our perovskite films showed smooth, uniform surfaces with full substrate coverage in scanning electron microscope (SEM) images (Figure 1b), which leads to good device performance and stability.

**Figure 1:**
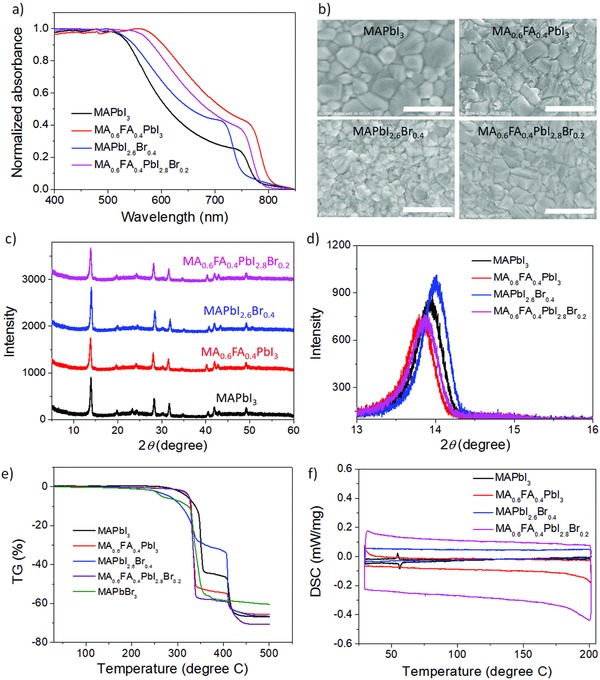
Optical, morphological, structural, and thermal properties of perovskite films with different compositions. a) Absorption spectra, b) SEM images with scale bars of 1 µm, and c,d) XRD patterns of perovskite films and e,f) TG and DSC properties of perovskite powders made from the precursor solutions used for PSC fabrication.

The X‐ray diffraction (XRD) patterns of four perovskite films shown in Figure 1c,d indicate that the FA and/or Br are indeed being integrated into the MAPbI_3_ crystal and forming MA_0.6_FA_0.4_PbI_3_, MAPbI_2.6_Br_0.4_, and MA_0.6_FA_0.4_PbI_2.8_Br_0.2_ alloys. A clear peak shift can be observed upon incorporating FA and/or Br, but all of the peaks can be indexed to the expected phases. The (110) diffraction peaks are slightly shifted to higher angles because of residual bromide while they move to lower angles when FA cations are added.^19^ No evidence of secondary phases or unincorporated PbI_2_ and organic cations was detected, which suggests that pure perovskite alloys were formed.

To understand the thermal properties of the perovskite‐alloy compounds, thermogravimetric analysis (TGA) and DSC were performed on MA_0.6_FA_0.4_PbI_3_, MAPbI_2.6_Br_0.4_, MA_0.6_FA_0.4_PbI_2.8_Br_0.2_, and MAPbI_3_ powders prepared by drying the precursor solutions used for the device fabrication at 100 °C for 30 min in a nitrogen‐filled glove box. The TGA results in Figure 1e show that MA_0.6_FA_0.4_PbI_3_ and MA_0.6_FA_0.4_PbI_2.8_Br_0.2_ have similar thermal decomposition temperatures while the decomposition temperature of MAPbI_3_ is as high as 300 °C. On the other hand, MAPbI_2.6_Br_0.4_ has a relatively low decomposition temperature of around 200 °C that is overlapped with pure MAPbBr_3_ (Olive line), which may be caused by phase separation of MAPbI_2.6_Br_0.4_ into MAPbI_3_ and MAPbBr_3_.^22^ At high temperatures, chemical decomposition and crystalline phase changes easily occur even in the case of the mixed halide perovskites.

Figure 1f displays DSC properties of the four perovskite powders. Similarly to the reported DSC properties of MAPbI_3_ single crystals,^16^, ^23^ a reversible phase transition between the tetragonal structure and the cubic structure was observed at 54.6 and 56.2 °C for the exothermic and endothermic processes, respectively. The other three perovskite alloys have no detectable phase transition between room temperature and 200 °C, suggesting that possible phase‐transition‐related degradation may not exist in the perovskite‐alloy‐based PSCs during device operation at temperatures around 60 °C, which is the normal surface temperature of solar cells under solar irradiation in high‐altitude areas.

Solar cells were fabricated by spin coating the perovskite alloys and MAPbI_3_ layers from precursor solutions, on top of glass substrates coated with a layer of indium tin oxide (ITO) and a layer of poly(3,4‐ethylenedioxythiophene)polystyrene sulfonate (PEDOT:PSS). To obtain uniform, flat perovskite films and similar device performance for unbiased comparison of the different compositions, we individually optimized spin‐coating conditions for each perovskite film. While the PEDOT:PSS layer was fabricated in air, all of the perovskite layers were fabricated in a nitrogen‐filled glove box to avoid any degradation in air. After deposition of C_60_ (30 nm), bathocuproine (BCP, 10 nm), and gold on the perovskite layers, all of the PSCs were encapsulated in the glove box with a glass cap and UV sealant before being removed for evaluation in ambient air. **Figure** **2**b shows the current density–voltage curves of the PSCs when operated under illumination from a solar simulator equipped with a Xenon lamp (AM1.5G, 100 mW cm^−2^). The values of short‐circuit current density (*J*_SC_), open‐circuit voltage (*V*_OC_), fill factor (FF), and power conversion efficiency (*η)* estimated from the current density–voltage curves are summarized in Table S1 in the Supporting Information.

**Figure 2:**
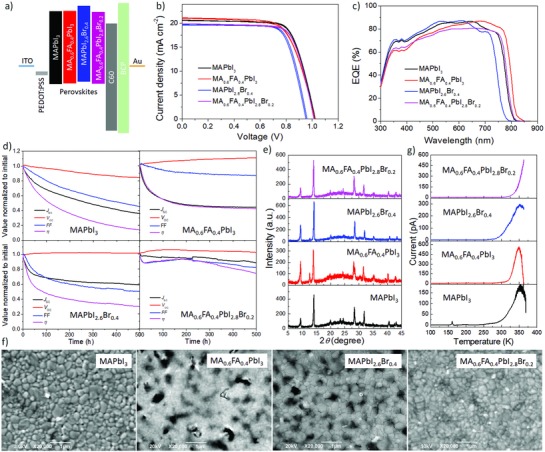
Device energy diagram, performance, lifetime, XRD, TSC, and SEM images of planar PSCs with different compositions. a) Energy diagram of the four perovskites along with the other materials used in the PSCs. b) Representative current density–voltage curves of the best PSC for each perovskite measured with forward (−0.2 to 1.2 V) and reverse (1.2 to −0.2 V) scans at a scan rate of 50 mV s^−1^ and with a delay time of 2 s. c) EQE spectra of four PSCs based on different perovskites. d) Typical evolution curves of *J*_SC_, *V*_OC_, FF, and η under continuous 1 sun solar irradiation (100 mW cm^−2^, AM 1.5G) at 85 °C under open‐circuit condition. To measure the evolution curves, each parameter was automatically measured every hour using a computer‐controlled sourcemeter. e–g) XRD, SEM, and TSC characterizations of PSCs aged for 500 h at 85 °C.

The CH_3_NH_3_PbI_3_‐based PSC exhibited *J*_SC_ = 20.93 mA cm^−2^, *V*_OC_ = 1.02 V, FF = 0.72, and η = 15.35% when measured under a forward bias scan. An enhanced *J*_SC_ of 21.31 mA cm^−2^ for the MA_0.6_FA_0.4_PbI_3_‐based PSC is attributed to a broader external quantum efficiency (EQE) spectrum as shown in Figure 2c, which is consistent with the red‐shifted absorption spectrum of MA_0.6_FA_0.4_PbI_3_. The *J*_SC_ (19.97 mA cm^−2^) was lower for the MAPbI_2.6_Br_0.4_‐based PSC because of the blue‐shifted absorption spectrum. For the MA_0.6_FA_0.4_PbI_2.8_Br_0.2_‐based PSC, we obtained *J*_SC_ = 20.14 mA cm^−2^, *V*_OC_ = 1.01 V, FF = 0.73, and η = 14.83%. All of the PSCs demonstrated comparable performance and negligible hysteresis because of their similar film morphologies and optical and electronic properties, which is important for reliable study and comparison of the device stability. Here, we would like to note that PSC efficiency is highly dependent on the fabrication environment and conditions. In some cases, for example, moisture in air can actually promote efficiency while having a detrimental effect on the lifetime. In our case, to obtain long device lifetimes, we strictly controlled the experimental conditions by fabricating in a glove box. On the other hand, typically, the invert structure based devices using PEDOT:PSS as hole transporting layer that we used always exhibit lower efficiencies than those of normal inorganic semiconductor, such as TiO_2_ and SnO_2_, based devices. Therefore, our device efficiency is still lower than those of reported champion devices. We have individually optimized the experiment conditions for all different perovskite‐based devices.

To discuss the influence of the perovskite composition on the stability without effects from UV‐induced perovskite degradation complicating the results, white light‐emitting diodes (WLEDs) were used as the light source. We first screened the high‐temperature stability of the four kinds of PSCs under continuous light irradiation of 500 h at 85 °C. Two regimes with different degradation speeds are observed in Figure 2d for all of the solar cells: an initial quick degradation within the first 100 h (namely, a burn‐in period), followed by a slow, relatively linear degradation. During the burn‐in period, *J*_SC_ and FF decreased while *V*_OC_ slightly increased in PSCs of perovskite alloys. The efficiency was reduced by only 12% during the burn‐in period for PSCs based on MA_0.6_FA_0.4_PbI_2.8_Br_0.2_, which is smaller than the nearly 50% decrease for the other PSCs after 100 h of high‐temperature operation. This phenomenon has been broadly observed in organic and polymer solar cells during device aging tests.^24^ This stage could be caused by stabilization of interface and morphology, which is different from case to case. After the burn‐in stage, the loss of η decelerates for the MAPbI_3_, MA_0.6_FA_0.4_PbI_3_, and MAPbI_2.6_Br_0.4_ PSCs. The MA_0.6_FA_0.4_PbI_2.8_Br_0.2_ PSC was the most stable, maintaining around 80% of its initial efficiency after 500 h, and exhibited a slight increase in η after burn‐in before then entering a linear degradation period. Note that the main decrease of η originates from a decrease of FF, which suggests that the efficiency and stability could be further improved by systematic interface engineering.

Figure 2e shows XRD patterns of the degraded samples. Strong diffraction peaks at 9.4° and weak diffraction peaks at 12.6° can be attributed to MAI and PbI_2_, respectively. These indicate that a larger amount of MAI was produced after degradation of all four types of perovskites at high temperature with continuous light irradiation.^25^ Because of protection imparted by the top metal electrode, the thermally volatile MAI could be kept inside the perovskite layers and detected by XRD after peeling off the metal electrode. Indeed, in our previous study, only a stronger diffraction peak for PbI_2_ was observed in a degraded device without encapsulation operated under room temperature.^12^ However, MAI might not be a key factor leading to degraded device performance because MAI similarly appeared in the degraded MA_0.6_FA_0.4_PbI_2.8_Br_0.2_ PSC, which had a much more stable η. This suggests that a certain amount of Schottky defects, such as MA^+^, Pb^2+^, and I^−^, are not seriously detrimental to long‐term stability of the PSCs.

SEM surface images (Figure 2f) of degraded PSCs were measured after peeling off the gold electrode. Some C_60_ and BCP might still remain on top of the perovskite because of stronger adhesion force between them than with the inorganic metal. The PSCs with mixed cations and anions showed significant morphological changes and the formation of large pin‐holes compared with the fresh films due to phase separation, and some small pin‐holes also appeared in the degraded MAPbI_3_ PSC. Only the MA_0.6_FA_0.4_PbI_2.8_Br_0.2_ film maintained full coverage.

In TSC profiles measured for PSCs degraded by 500 h of illumination at 85 °C (Figure 2g), carrier traps were not detected in the degraded MA_0.6_FA_0.4_PbI_2.8_Br_0.2_ PSC, which would be consistent with it having the best device stability. In the other two aged MA_0.6_FA_0.4_PbI_3_, and MAPbI_2.6_Br_0.4_ devices, similar TSC peaks were observed at approximately 350 K that could be ascribed to the traps caused by interface deterioration between perovskite and the cathode, which is consistent with significant morphological changes and the formation of pin‐holes as shown in SEM images.^26^ Note that no detectable carrier traps were found in the high‐performance fresh devices, and also no such phase‐transition‐related traps were observed in the fresh and aged devices when the stability test was performed at room temperature, as was previously reported.^12^ Furthermore, we found that mesoporous‐TiO_2_‐based PSCs produced complex TSC signals with bad reproducibility. Use of inverted device structures with PEDOT:PSS as hole‐transporting layer is important to obtain reliable TSC results.^27^

The TSC profile of MAPbI_3_ PSC was more complex. Two TSC peaks were observed at 161 and 201 K, indicating the formation of two kinds of carrier traps with corresponding trap depths of 0.32 and 0.41 eV, respectively. The presence of carrier traps related to the low‐temperature phase transition is consistent with previous reports.^11^, ^12^ However, no detectable signals were observed in this temperature range in the degraded PSCs based on perovskite alloys (see Figure S1, Supporting Information), confirming that the use of perovskite alloys can suppress the formation of shallow carrier traps under continuous light irradiation. Moreover, a large number of deep traps at temperatures over 300 K, which covers the high‐temperature phase transition (around 330 K) from the tetragonal to cubic phase, are generated in the degraded MAPbI_3_ PSC. This proves that these carrier traps are caused by continuous heating and light irradiation are detrimental to device stability.^11^, ^28^ We believe that high‐temperature phase transition from tetragonal to cubic could accelerate defect formation around grain boundaries through changes in the crystalline lattice.

Similar TSC curves were observed in the degraded MAPbI_2.6_Br_0.4_ device, which had a degradation behavior similar to that of the MAPbI_3_ PSC as shown in Figure 2d. This can be ascribed to the formation of pure phase of semicrystalline MAPbI_3_ due to phase separation of MAPbI_2.6_Br_0.4_, which has already be observed in a previous study.^22^ These results show that morphological changes in addition to the formation of carrier traps occur at high temperatures, providing an additional reason why MAPbI_3_‐based devices are unstable under high temperature. Note that we repeated all degradation experiments and other characterization at least three times to confirm the reliability of the results.

To further understand the relationship between phase transition and device degradation, we selected two perovskite‐ based devices for electrochemical impedance spectroscopy (EIS) measurements,^29^, ^30^ fresh and aged for 500 h MAPbI_3_ and MA_0.6_FA_0.4_PbI_2.8_Br_0.2_ devices, respectively. EIS was performed at 25, 55, and 70 °C during heating and again at the same temperatures during cooling down. A waiting time of 10 min was inserted prior to each measurement to ensure thermal equilibrium. It is noteworthy that we also measured current density–voltage curves before and after each EIS. The parameters FF, η, *J*_sc_, and *V*_oc_ did not show any distinct trend during these tests, and device performance was not affected by the EIS measurements. The obtained spectra were fitted to a simple process‐oriented equivalent circuit model (ECM)^31^ as shown in **Figure** **3**a. This might be a significant simplification, but we think it is appropriate for this qualitative analysis. Moreover, it has been shown that the values determined with this circuit are comparable to other models,^32^ such as the basic model shown in ref. 28. None of the spectra measured in this study showed negative loops.^33^ The spectra measured initially at 25 °C and at 70 °C for fresh and aged MAPbI_3_ and MA_0.6_FA_0.4_PbI_2.8_Br_0.2_ devices are shown in Figure 3b–e, respectively. These spectra demonstrate best the impact of the phase transition in MAPbI_3_ because 70 °C is distinctly above the phase equilibrium temperature. The evolution of the resistances *R*_0_, *R*_1_, and *R*_2_ for all measurements is plotted in Figure 3f–h. We also calculated the effective capacities, *C*_1_ and *C*_2_, from the respective resistances, *R_x_*, and time constants, *τ_x_*, of the RQ subcircuits (parallel connection of a resistor and a constant phase element): *C_x_* = *τ_x_*/*R_x_*. These are shown in Figure 3i.

**Figure 3:**
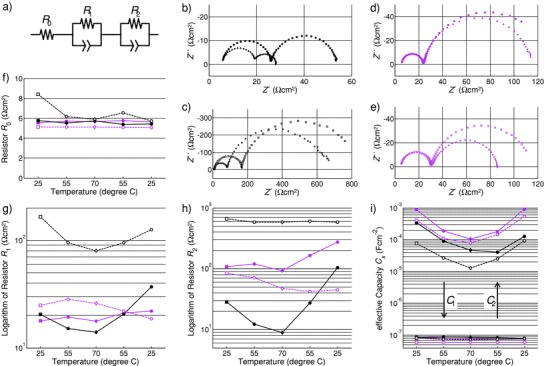
Impedance analysis of the devices. a) Process‐oriented ECM to which the spectra were fitted. b–e) Selected impedance spectra showing the initial measurement at 25 °C (squares) and at the maximum temperature at 70 °C (diamonds) on the fresh and aged MAPbI_3_ devices (black), fresh and aged MA_0.6_FA_0.4_PbI_2.8_Br_0.2_ devices (magenta). f–i) Evolution of the resistances *R*_0_, *R*_1_, and *R*_2_ determined by ECM fit from spectra measured during heating up at 25, 55, and 70 °C and subsequently cooling down to 55 and 25 °C again. Note that the color code is maintained throughout this figure. Fresh samples are indicated by full symbols and lines, aged samples by empty symbols and dashed lines. The square and diamond symbols of (b)–(e) are also indicated in (f)–(i).

The ohmic resistances, *R*_0_, for all samples lie between 5.04 and 5.73 Ω cm² after the temperature treatment (Figure 3f), so we exclude a serious contact loss due to morphology changes as reason for the degradation of the aged MAPbI_3_ device. Further, it is apparent that both the fresh and the aged MA_0.6_FA_0.4_PbI_2.8_Br_0.2_ devices do not show any change in the high‐frequency arc due to the increased temperature, whereas fresh and aged MAPbI_3_ devices exhibit a significant decrease of its diameter at 70 °C. There is still some discussion about the origin of this process.^33^, ^34^ However, *R*_1_ shows a distinct dependency on the temperature for MAPbI_3_ devices and it was mainly attributed to the bulk perovskite in the past.^33^ It could be associated with carrier traps that are removed beyond the phase equilibrium temperature. Repeated cycling probably increases the overall number of these traps, which would explain the tenfold increase of *R*_1_ for the aged MAPbI_3_ device. The related capacity *C*_1_ exhibits values <10^−7^ F cm^−2^ for all measured spectra, which suggests that it is a geometrical capacitance. The good agreement of all values for *C*_1_ is another strong indication that the degradation of the aged MAPbI_3_ device was not caused by morphological changes, which are expected to come along with a significant decrease of *C*_1_.

Another noticeable feature in this series is the reduction of *R*_2_ of the fresh MAPbI_3_ device during the first heating, from 28 Ω cm^2^ at 25 °C to 9 Ω cm^2^ at 70 °C, indicating a serious change in MAPbI_3_ device whereas *R*_2_ for MA_0.6_FA_0.4_PbI_2.8_Br_0.2_ device is almost unaffected by the first heat treatment. However, all fresh samples show an increase in *R*_2_ after cooling down, which we attribute to the state before device recovery, as will be discussed below. *R*_2_ has been attributed to the interfaces^33^ or a coupled electronic‐ionic impedance,^34^ where the distribution of ions strongly influences charge carrier recombination, which in turn would be expected to happen predominantly at the interfaces. *C*_2_ on the other hand shows a distinct temperature dependency for all samples, which indicates that its value does not change due to the phase transition. For the aged samples *C*_2_ shows a similar minor decrease, which is why we do not see it as very indicative for the current study and do not want to speculate about its origin and behavior at this point.

Concluding the results of the impedance analysis, it is unlikely that the following observations are caused by a morphology change: i) the 68.6% drop in *R*_2_ for the fresh MAPbI_3_ device at 70 C, and ii) the increase of *R*_2_ for MAPbI_3_ by almost two orders of magnitude after ageing without affecting *R*_0_ or *C*_1_. Rather, we see these results as a further indication that the phase transformation during heat treatment of the MAPbI_3_ device introduces additional carrier traps and that these are the main reason for the reduced thermal stability of this material.

It is obvious from the impedance analysis of fresh and aged devices that MAPbI_3_ shows a distinct degradation upon temperature cycling already for one thermal cycle as well as for the device after the 500 h aging test, whereas the MA_0.6_FA_0.4_PbI_2.8_Br_0.2_ device is robust against heat treatments up to 70 °C for all tests discussed in this section. All model parameters and the residuals for the fits are provided in Figure S2 and Table S2 in the Supporting Information.

According to the earlier discussion of device stability and EIS, a reduction of carrier traps will contribute to improving the device stability. To further confirm the thermal stability of the perovskite alloy and the influence of the phase transition, we performed thermal cycling tests using standard ISOS‐T‐1 thermal cycling.^17^ **Figure** **4**a,b shows the evolution curves of MA_0.6_FA_0.4_PbI_2.8_Br_0.2_‐ and MAPbI_3_‐based devices during thermal cycling between 25 and 85 °C (four cycles) under continuous light irradiation. At first, MAPbI_3_‐based device was extremely stable under the initial test period at 25 °C, indicating high‐quality devices.^15^ When the device temperature was initially raised to 85 °C, η dropped around 7% compared with that at 25 °C because of a decrease in *V*_OC_. This loss in *V*_OC_ is consistent with previous results and could be caused by increased recombination.^9^ Similar with previous result, MA_0.6_FA_0.4_PbI_2.8_Br_0.2_ device underwent a burn‐in period at both 25 and 85 °C. As the devices continued to operate at 85 °C, efficiency only slightly reduced after burn‐in period in the case of the MA_0.6_FA_0.4_PbI_2.8_Br_0.2_ device. On the contrary, the performance of the MAPbI_3_‐based device quickly decreased because of a reduction in *J*_SC_, which is similar to the previous results. When the temperature returned to 25 °C, the device performance of MA_0.6_FA_0.4_PbI_2.8_Br_0.2_ experienced a vibration and decrease stage, owing to a recovery of the FF, and then kept stable. However, the efficiency of the MAPbI_3_ PSC did not recover as much because of a significant drop in FF despite a recovery of *V*_OC_.

**Figure 4:**
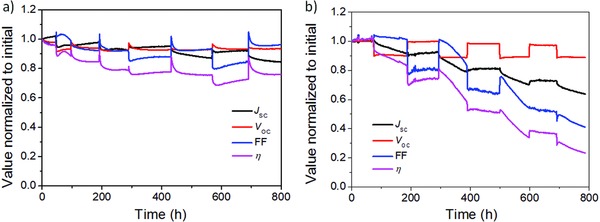
Device lifetime characterization with thermal cycling. Typical evolution curves of a) *J*_SC_, *V*_OC_, FF, and η of MA_0.6_FA_0.4_PbI_2.8_Br_0.2_ and b) MAPbI_3_ under continuous 1 sun solar irradiation (100 mW cm^−2^, AM 1.5G) with thermal cycling between 25 and 85 °C according to the ISOS‐T‐1 standard under open‐circuit condition.

The degradation of MAPbI_3_ device is further seen in the second high‐temperature operation region, during which the *J*sc, FF, and η continuously and greatly decrease. The reduction of *J*sc and FF means increase of carrier traps. As a comparison, MA_0.6_FA_0.4_PbI_2.8_Br_0.2_‐based device still showed similar behavior as during the first cycle and with only a small amount of degradation. In the latter two cycles, the MA_0.6_FA_0.4_PbI_2.8_Br_0.2_ device became more stable. After all four cycles, its η remained at 80% of its initial value. In contrast, η of the MAPbI_3_ PSC maintained only 23% of its initial η.

In conclusion, we studied the degradation behavior of four different perovskite‐based solar cells. Large carrier trap densities were observed in the TSC curve of MAPbI_3_‐based PSCs aged under high operating temperatures. These carrier traps are detrimental to long‐term stability. Perovskite alloys with mixed both cations and anions could effectively avoid the formation of phase‐transition‐induced carrier traps because of a lack of a similar intrinsic phase transition. The EIS of fresh and aged devices under different temperature further confirmed effects of the phase transition of MAPbI_3_ on the formation of carrier traps and degradation. Finally, PSCs with improved thermal stability were demonstrated. We believe that the present findings offer insight to help obtain efficient and stable organic–inorganic hybrid PSCs for future applications.

## Experimental Section

*Device Fabrication and Characterization*: Glass substrates coated with prepatterned ITO layers with a thickness of ≈150 nm (ATSUGI MICRO) and sheet resistance of 12 Ω sq.^−1^ were cleaned sequentially by ultrasonicating in a detergent solution, pure water, acetone, and isopropanol for 10 min each and then subjected to UV–ozone treatment for 15 min. A thin layer (≈50 nm) of PEDOT:PSS (Clevios, Al4083) was prepared by spin coating at 3000 rpm for 45 s on top of ITO in air using a poly(tetrafluoroethylene) syringe filter with a 0.45 µm pore diameter, followed by baking the PEDOT:PSS layer at 160 °C for 10 min. The perovskite layer was prepared in a nitrogen‐filled glove box (H_2_O and O_2_ concentrations <0.1 ppm) using a one‐step method in the following way. A mixture of PbI_2_ (98%; TCI) and CH_3_NH_3_I (1:1 by mol) for CH_3_NH_3_PbI_3_ and mixtures with certain ratio of PbI_2_, PbBr_2_ (98%; TCI), CH_3_NH_3_I (TCI), and HC(NH_2_)_2_I (TCI) for perovskite alloys were dissolved in mixtures of γ‐butyrolactone (GBL) and DMF (4:6 vol/vol; GBL, ≥99%; DMF, 99.8%; TCI) at 1.2 m and stirred at 60 °C for 12 h. The mixtures were then spin‐coated on the PEDOT:PSS layer at 4000 rpm for 30 s. During spin coating, 0.3 mL of toluene was dropped onto the perovskite precursor layer. The precursor layer was baked on a hotplate at 60 °C for 15 min, followed by 100 °C for 30 min. The thickness of the perovskite layer was measured to be around 300 nm using a Dektak profilometer (DektakXT, Bruker). Finally, 30 nm C_60_, 10 nm BCP, and 100 nm Au layers were thermally deposited on top of the CH_3_NH_3_PbI_3_ layer under a high vacuum (10^−4^ Pa) through a contact shadow mask. After unloading the PSCs directly into a glove box attached to the evaporation system, the PSCs were encapsulated using a glass lid and UV‐cured sealant. Current density–voltage and EQE measurements were performed on the PSCs using a computer‐controlled Keithley 2400 source unit and an EQE measurement system (WXS‐155S‐10: Wacom Denso) under simulated AM1.5G solar illumination from a Xe lamp‐based solar simulator (SRO‐25 GD, Bunkokeiki). The active area of the PSC was defined to be 16 mm^2^ by the overlap of the patterned ITO and Au electrodes. The lamp power was carefully calibrated at 100 mW cm^−2^ (1 sun) using a crystalline Si reference cell with an amorphous Si optical filter (Bunkokeiki), which was certificated by the National Institute of Advanced Industrial Science and Technology of Japan. The photovoltaic performance of the devices was not confirmed from independent certification laboratories.

For characterization of degraded perovskites, such as XRD and SEM, scotch tape was used to peel off the top metal electrode after carefully removing the encapsulation glass.

*Device Lifetime Measurements*: Stimulated solar light (AM1.5G) from WLEDs was continuously illuminated onto the PSCs at open‐circuit conditions with the devices held at 25 or 85 °C by a temperature controller. Time‐dependent *V*_OC_, *J*_SC_, FF, and η were measured automatically using a lifetime measurement system (System Engineers). For thermal cycling tests, the device temperature was changed by hand approximately every hundred hours.

*TSC Measurement*: The PSC was placed in a TSC measurement chamber (Rigaku TSC‐FETT EL2000), and the ITO anode and Au cathode layers were connected to gold leads. The chamber was then evacuated using a rotary mechanical pump and filled with helium, which acted as a heat transfer medium. These evacuation and filling procedures were repeated three times to completely replace the atmosphere in the chamber with helium. The device was cooled to −183 °C (90 K) using liquid nitrogen. The PSC was biased at 1 mA cm^−2^ for 2 min at liquid nitrogen temperatures to fill carrier traps with injected carriers from the electrodes. The device temperature was then increased up to 110 °C (383 K) at a heating rate of 5 K min^−1^. The carriers released from the traps during the heating process were measured as current to draw the TSC curves. The background current curve was measured without trap filling at the liquid nitrogen temperature. The trap depth (*E*_T_) could be calculated using Equation (1).

(1) $$E_{T}=k_{B}T_{m}\ln\left(T_{m}{}^{4}/\beta \right)$$

where *k*_B_ is Bolzmann's constant (8.617 × 10^−5^ eV K^−1^), *T*_m_ is the temperature of the TSC peak, and β is the heating rate (5 K min^−1^).

The trap density (*N*_t_) is given by Equation (2).

(2) $$N_{t}=Q/qAL$$

where *Q* is the area under the TSC peak, which is equal to the number of charges (in this case, holes) emitted from the sample during the heating process, *q* is the electronic charge, *A* is the active device area, and *L* is the layer thickness.

*Absorption Measurement*: Ultraviolet–visible–near‐infrared absorption spectra of the perovskite films were measured using a Perkin‐Elmer Lambda 950‐PKA spectrophotometer in air with a relative humidity of 25%. An ITO‐coated glass substrate was used as a reference.

*XRD Measurement*: The XRD characteristics were evaluated with an XRD system using a 2 θ/θ technique (λ = 1.54 Å (CuKα)) (Rigaku, RINT‐2500). Diffraction peaks coming from ITO, PEDOT:PSS, and C_60_ were undetectable.

*Impedance Measurements*: Impedance spectra were measured with a Solartron 1260 frequency response analyzer in the range of 1 MHz to 700 mHz. All measurements were performed at a voltage bias of 0.6 V under illumination of stimulated solar light (AM1.5G) from WLEDs and with an amplitude of 20 mV. To improve the data quality at high frequencies, the internal high‐pass filter was applied beyond 1 kHz.

## Conflict of Interest

The authors declare no conflict of interest.

## Supporting information

Supplementary
