# Supplementary material for: The Relation of Phase‐Transition Effects and Thermal Stability of Planar Perovskite Solar Cells
Source: Adv Sci (Weinh). 2018 Oct 25;6(1):1801079. doi: 10.1002/advs.201801079 (PMC6325570; doi:10.1002/advs.201801079)
Supplement: Supplementary file 1 — Supplementary [file ADVS-6-1801079-s001.pdf]

Copyright WILEY-VCH Verlag GmbH & Co. KGaA, 69469 Weinheim, Germany, 2018.

ADVANCED SCIENCE
Open Access

Supporting Information

for *Adv. Sci.,* DOI: 10.1002/advs.201801079

The Relation of Phase-Transition Effects and Thermal Stability of Planar Perovskite Solar Cells

Chuanjiang Qin, Toshinori Matsushima, Dino Klotz, Takashi Fujihara, and Chihaya Adachi**

Supplementary Information

The relation of phase transition effects and thermal stability of planar perovskite solar cells

Chuanjiang Qin,[1,2] Toshinori Matsushima,[1,2,3] Dino Klotz,[3] Takashi Fujihara,[4] and Chihaya Adachi[1,2,3,4]

[1]Center for Organic Photonics and Electronics Research (OPERA), Kyushu University, 744 Motooka, Nishi, Fukuoka 819-0395, Japan

[2]Japan Science and Technology Agency (JST), ERATO, Adachi Molecular Exciton Engineering Project, 744 Motooka, Nishi, Fukuoka 819-0395, Japan

[3]International Institute for Carbon Neutral Energy Research (WPI-I^{2}CNER), Kyushu University 744 Motooka, Nishi, Fukuoka 819-0395, Japan

[4]Innovative Organic Device Laboratory, Institute of Systems, Information Technologies and Nanotechnologies (ISIT), 744 Motooka, Nishi, Fukuoka 819-0395, Japan

Correspondence and requests for materials should be addressed to C.A. (email: adachi@cstf.kyushu-u.ac.jp) or to C.Q. (email: cjqin@opera.kyushu-u.ac.jp)

Table S1. Statistics on solar cell performance of the PSCs using different perovskites. Average values and standard deviations were calculated for 20 cells in 3 different batches of PSCs fabricated under identical conditions.

Device	J_{SC} / mA cm^{-2}	V_{OC} / V	FF	η / %
**$MA_{0.6}FA_{0.4}PbI_3$**	21.15 ± 0.22	1.02 ± 0.01	0.67 ± 0.03	14.26 ± 0.32
**$MAPbI_{2.6}Br_{0.4}$**	19.88 ± 0.26	0.95 ± 0.02	0.72 ± 0.03	13.95 ± 0.21
**$MA_{0.6}FA_{0.4}PbI_{2.8}Br_{0.2}$**	20.21 ± 0.32	1.01 ± 0.01	0.71 ± 0.02	14.53 ± 0.22
**$MAPbI_3$**	20.51 ± 0.28	1.02 ± 0.01	0.71 ± 0.03	15.15 ± 0.28

Table S2. Fit parameters obtained from the impedance analysis. The equivalent circuit used is displayed in Figure 3a in the main article. Listed are the resistances, R_x, the time constants, τ_x, and the exponents, n_x, for the constant phase elements. The capacitances, C_x, are the effective capacitances calculated by $C_x = \tau_x / R_x$.

$MAPbI_3$ (fresh)	R_0 (Ωcm²)	R_1 (Ωcm²)	τ_1 (s)	C_1 (Fcm^{-2})	n_1	R_2 (Ωcm²)	τ_2 (s)	C_2 (Fcm^{-2})	n_2
#1 (25°C)	5.77	20.42	$1.73 \cdot 10^{-6}$	$8.46 \cdot 10^{-8}$	0.98	28.42	$9.82 \cdot 10^{-3}$	$3.45 \cdot 10^{-4}$	0.88
#2 (55°C)	5.53	15.11	$1.33 \cdot 10^{-6}$	$8.79 \cdot 10^{-8}$	0.98	12.24	$1.11 \cdot 10^{-3}$	$9.04 \cdot 10^{-5}$	0.93
#3 (70°C)	5.73	13.96	$1.20 \cdot 10^{-6}$	$8.63 \cdot 10^{-8}$	0.98	8.91	$4.19 \cdot 10^{-4}$	$4.70 \cdot 10^{-5}$	0.97
#4 (55°C)	5.40	20.68	$1.72 \cdot 10^{-6}$	$8.34 \cdot 10^{-8}$	0.98	27.39	$1.13 \cdot 10^{-3}$	$4.12 \cdot 10^{-5}$	0.89
#5 (25°C)	5.41	36.89	$2.94 \cdot 10^{-6}$	$7.97 \cdot 10^{-8}$	0.98	103.73	$1.31 \cdot 10^{-2}$	$1.27 \cdot 10^{-4}$	0.85

$MAPbI_3$ (aged)	R_0 (Ωcm²)	R_1 (Ωcm²)	τ_1 (s)	C_1 (Fcm^{-2})	n_1	R_2 (Ωcm²)	τ_1 (s)	C_2 (Fcm^{-2})	n_2
#1 (25°C)	8.41	165.35	$1.37 \cdot 10^{-5}$	$8.28 \cdot 10^{-8}$	0.96	662.12	$5.05 \cdot 10^{-2}$	$7.63 \cdot 10^{-5}$	0.89
#2 (55°C)	6.15	94.82	$7.05 \cdot 10^{-6}$	$7.43 \cdot 10^{-8}$	0.96	580.94	$1.56 \cdot 10^{-2}$	$2.68 \cdot 10^{-5}$	0.88
#3 (70°C)	5.89	79.88	$5.79 \cdot 10^{-6}$	$7.25 \cdot 10^{-8}$	0.96	582.19	$7.62 \cdot 10^{-3}$	$1.31 \cdot 10^{-5}$	0.86
#4 (55°C)	6.53	95.11	$7.01 \cdot 10^{-6}$	$7.37 \cdot 10^{-8}$	0.97	602.52	$1.49 \cdot 10^{-2}$	$2.47 \cdot 10^{-5}$	0.88
#5 (25°C)	5.73	125.34	$9.68 \cdot 10^{-6}$	$7.73 \cdot 10^{-8}$	0.96	581.30	$5.35 \cdot 10^{-2}$	$9.21 \cdot 10^{-5}$	0.91

$MA_{0.6}FA_{0.4}PbI_{2.8}Br_{0.2}$ (fresh)	R_0 (Ωcm²)	R_1 (Ωcm²)	τ_1 (s)	C_1 (Fcm^{-2})	n_1	R_2 (Ωcm²)	τ_1 (s)	C_2 (Fcm^{-2})	n_2
#1 (25°C)	5.57	17.81	$1.55 \cdot 10^{-6}$	$8.70 \cdot 10^{-8}$	0.98	108.03	$9.51 \cdot 10^{-2}$	$8.80 \cdot 10^{-4}$	0.87
#2 (55°C)	5.69	19.35	$1.56 \cdot 10^{-6}$	$8.08 \cdot 10^{-8}$	0.99	120.65	$2.30 \cdot 10^{-2}$	$1.90 \cdot 10^{-4}$	0.88
#3 (70°C)	5.64	17.71	$1.40 \cdot 10^{-6}$	$7.90 \cdot 10^{-8}$	0.98	92.74	$9.79 \cdot 10^{-3}$	$1.06 \cdot 10^{-4}$	0.88
#4 (55°C)	5.77	21.10	$1.59 \cdot 10^{-6}$	$7.52 \cdot 10^{-8}$	0.98	163.99	$2.94 \cdot 10^{-2}$	$1.79 \cdot 10^{-4}$	0.84
#5 (25°C)	5.62	22.03	$1.67 \cdot 10^{-6}$	$7.58 \cdot 10^{-8}$	0.97	276.10	$2.54 \cdot 10^{-1}$	$9.22 \cdot 10^{-4}$	0.77

$MA_{0.6}FA_{0.4}PbI_{2.8}Br_{0.2}$ (aged)	R_0 (Ωcm²)	R_1 (Ωcm²)	τ_1 (s)	C_1 (Fcm^{-2})	n_1	R_2 (Ωcm²)	τ_1 (s)	C_2 (Fcm^{-2})	n_2
#1 (25°C)	5.11	24.81	$1.49 \cdot 10^{-6}$	$6.02 \cdot 10^{-8}$	0.98	85.08	$3.48 \cdot 10^{-2}$	$4.10 \cdot 10^{-4}$	0.87
#2 (55°C)	5.12	28.32	$1.67 \cdot 10^{-6}$	$5.91 \cdot 10^{-8}$	0.98	71.29	$7.94 \cdot 10^{-3}$	$1.11 \cdot 10^{-4}$	0.92
#3 (70°C)	5.13	25.90	$1.54 \cdot 10^{-6}$	$5.95 \cdot 10^{-8}$	0.97	47.16	$3.71 \cdot 10^{-3}$	$7.86 \cdot 10^{-5}$	0.98
#4 (55°C)	5.09	21.96	$1.30 \cdot 10^{-6}$	$5.94 \cdot 10^{-8}$	0.98	42.06	$6.13 \cdot 10^{-3}$	$1.46 \cdot 10^{-4}$	0.93
#5 (25°C)	5.04	18.62	$1.11 \cdot 10^{-6}$	$5.98 \cdot 10^{-8}$	0.97	44.72	$2.40 \cdot 10^{-2}$	$5.38 \cdot 10^{-4}$	0.86

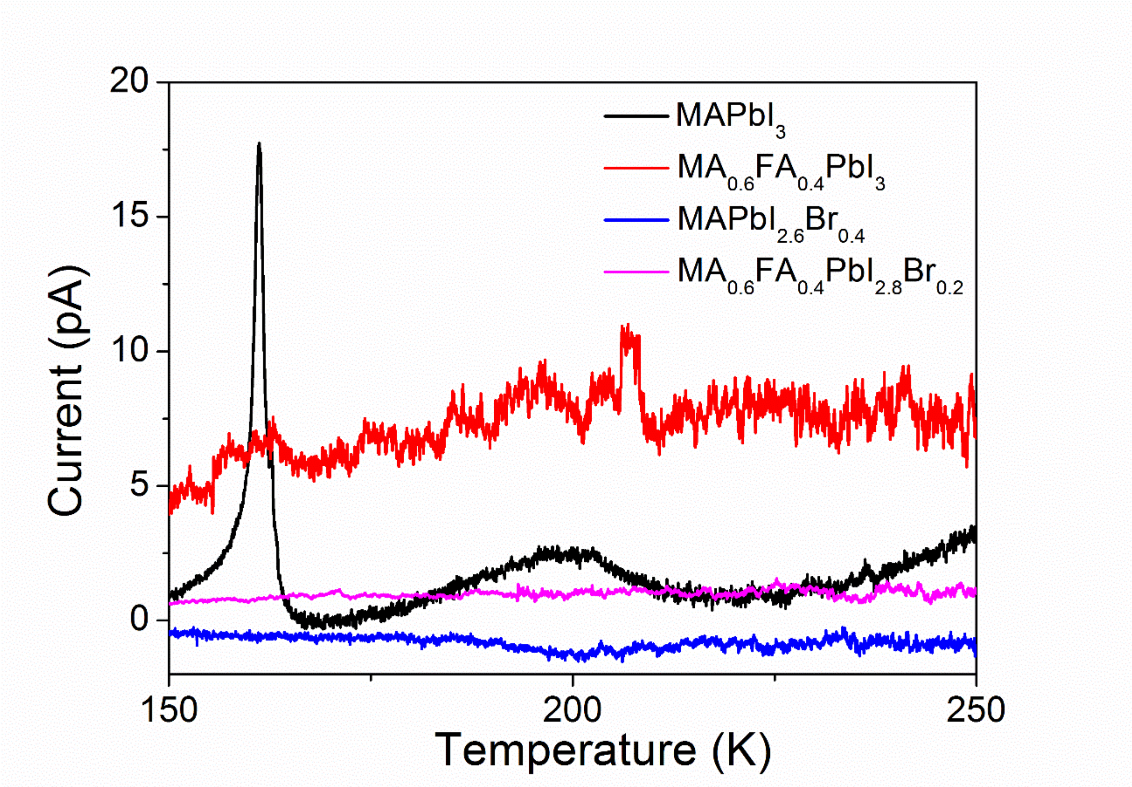

Figure S1. TSC curves in the low-temperature region for PSCs aged after 500 h at 85 °C.

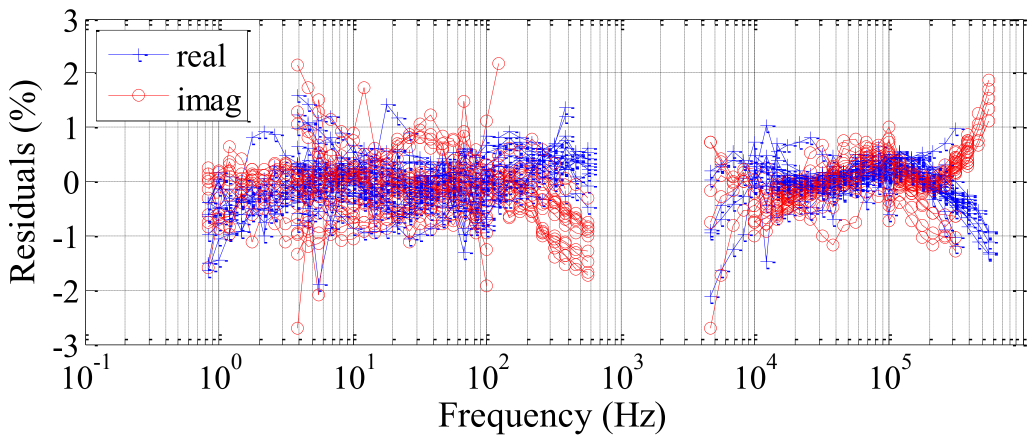

Figure S2. Residuals for the impedance fits. In this figure, all residuals for the fits that lead to the parameters in Table S2 are overlaid in one diagram. Note that the fit was performed in two stages because also the impedance spectra were recorded in two steps in order to be able to use optimized settings for the measurement at high and low frequencies.

All residuals are below 3% with the majority being below 1%, which proves the accuracy of the results presented in this study.
